# Apolipoprotein E in VLDL and LDL With Apolipoprotein C‐III is Associated With a Lower Risk of Coronary Heart Disease

**DOI:** 10.1161/JAHA.113.000130

**Published:** 2013-06-21

**Authors:** Carlos O. Mendivil, Eric B. Rimm, Jeremy Furtado, Frank M. Sacks

**Affiliations:** 1 Universidad de los Andes, School of Medicine, Bogotá, Colombia (C.O.M.); 2 Department of Nutrition, Harvard School of Public Health, Boston, MA (C.O.M., E.B.R., J.F., F.M.S.); 3 Department of Epidemiology, Harvard School of Public Health, Boston, MA (E.B.R.); 4 Channing Laboratory, Department of Medicine, Brigham and Women's Hospital and Harvard Medical School, Boston, MA (E.B.R., F.M.S.)

**Keywords:** apolipoprotein, cholesterol, coronary disease, lipoproteins, risk factor

## Abstract

**Background:**

Low‐density lipoprotein (LDL) with apolipoprotein C‐III (apoC‐III) is the lipoprotein species that most strongly predicts initial and recurring coronary heart disease (CHD) events in several cohorts. Thus, a large portion of the CHD risk conferred by LDL may be attributable to LDL that contains apoC‐III. Very‐low‐density lipoprotein (VLDL) and LDL with apoC‐III have varying amounts of apoE. We hypothesized that a high content of apoE lessens the adverse influence of apoC‐III on the risk of CHD because it promotes the clearance of VLDL and LDL from plasma.

**Methods and Results:**

We studied 2 independent cohorts, the Nurses' Health Study, composed of women, and the Health Professionals Follow‐up Study, composed of men. These cohorts contributed to this study 322 women and 418 men initially free of CVD who developed a fatal or nonfatal myocardial infarction during 10 to 14 years of follow‐up and matched controls who remained free of CHD. The apoE content of LDL with apoC‐III was inversely associated with CHD after multivariable adjustment (relative risk for top versus bottom quintile 0.53, 95% CI 0.35 to 0.80). The apoE content of VLDL with apoC‐III had a similar inverse association with CHD. The highest risks were associated with a high apoB concentration and a low apoE content of LDL with apoC‐III or of VLDL+LDL with apoC‐III. The observed associations were in both male and female cohorts and independent of traditional CHD risk factors and of C‐reactive protein.

**Conclusions:**

An increased apoE content in VLDL and LDL with apoC‐III was associated with a lower risk of CHD. Strategies to enrich VLDL and LDL in apoE are worth exploring for the prevention of CHD.

## Introduction

Apolipoprotein E (apoE) is a small apolipoprotein synthesized mostly by the liver that serves as a ligand to the low‐density lipoprotein (LDL) receptor (LDLR) and the LDL‐receptor–related protein‐1 (LRP1) and plays an essential role in metabolism by promoting cellular uptake of lipoproteins.^1–2^ Even though most plasma apoE is borne in HDL and VLDL, there is a measurable concentration of apoE in LDL that is distributed throughout its conventional density range that includes the intermediate‐density lipoproteins (IDL) and light and dense LDL.^3–4^ The usual plasma concentration of apoE ranges between 6 and 10 mg/dL,^5^ of which 25% to 50% is associated with VLDL, 10% to 25% with IDL/LDL, and 30% to 50% with HDL. apoE has a very high affinity for the LDL receptor, actually much superior to that of apoB‐100^6^; hence, apoE in VLDL and LDL may influence the plasma concentration and metabolic destination of these lipoproteins, with potential implications for atherogenesis and the occurrence of cardiovascular disease (CVD). In addition, apoE has diverse proposed antiatherogenic properties independent of its role on lipoprotein uptake.^7^

Even though genetic variations at the *APOE* gene have been extensively studied as predictors of cardiovascular events,^8–10^ very few studies have analyzed concentrations of the apoE protein as predictors of cardiovascular outcomes or how they are affected by concentrations of apolipoprotein C‐III (apoC‐III). One study in adults older than 85 years^11^ found a positive association between total plasma apoE concentrations and cardiovascular mortality, and another reported a generally increased risk of stroke with higher plasma apoE in the same cohort of older individuals.^12^ However, plasma total apoE is distributed among all lipoprotein classes, and a considerable amount of apoE is in HDL. The apoE content of HDL is associated with recurrent coronary heart disease (CHD) events.^13^ Thus, the influence of apoE on atherosclerosis and CHD may depend on the lipoprotein where it is located.

**Figure 1.**
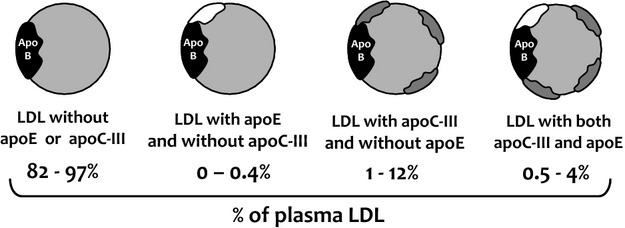
Relative distribution of the plasma LDL subpopulations according to contents of apoE and apoC‐III.^3–4^ Data expressed as percentage of total LDL apoB concentration. LDL indicates low‐density lipoprotein; apoE, apolipoprotein E; apoC‐III, apolipoprotein C‐III.

ApoC‐III is another small apolipoprotein with metabolic actions antagonistic to those of apoE; apoC‐III impedes binding of VLDL to cellular receptors, channeling the metabolism of VLDL away from clearance from the circulation and toward conversion to LDL, especially dense LDL.^14–15^ apoC‐III also specifically and directly induces proatherogenic changes in monocytes and endothelial cells.^16^ The plasma concentration of apoC‐III in VLDL and LDL is associated with CHD in retrospective studies^17–18^ and predicts initial and recurrent CHD in prospective studies.^13^ Among the concentrations of the apoB lipoproteins, the plasma concentration of LDL with apoC‐III predicts most strongly the incidence of recurrent cardiovascular events in type 2 diabetes^19^ and first coronary event in 2 US cohorts initially free of CHD.^20^ The relative risk of a recurrent event in the cohort with type 2 diabetes was 6.2 for the fifth versus first quintile of LDL with apoC‐III compared with 2.2 for the fifth versus first quintile of LDL without apoC‐III. In the 2 US cohorts of men and women, the relative risk of a first coronary event was 2.4 for LDL with apoC‐III compared with 1.2 for LDL without apoC‐III,^20^ and the test for interaction was significant demonstrating that the prediction of CHD risk by the 2 types of LDL differed significantly. Interestingly, about 30% of LDL that has apoC‐III also has apoE.^3–4^ It could be that the balance between the apoE and apoC‐III contents of an individual's LDL affects the physiology of LDL and, subsequently, the progression of atherosclerosis and the occurrence of a coronary event. On the other hand, since nearly all of the apoE in LDL is in LDL with apoC‐III^3–4^ (Figure 1), it follows that the influence of apoE needs to be studied in the context of apoC‐III–containing LDL, and in this study we have focused our study on apoE in LDL that also contains apoC‐III.

We explored whether the apoE content of LDL and VLDL with apoC‐III (expressed as the molar ratio of apoE to apoB in these particles) may be associated with an attenuated CHD risk. We prospectively studied this relationship in 2 large US cohorts of women and men, the Nurses' Health Study (NHS) and the Health Professionals Follow‐up Study (HPFS), separately and combined.

## Methods

### Study Population

The NHS and the HPFS are prospective cohort investigations respectively involving 121 700 female US registered nurses who were 30 to 55 years old at baseline in 1976 and 51 529 US male health professionals who were 40 to 75 years old at baseline in 1986. Information about health and disease is assessed biennially.^21–24^

From 1989 through 1990, a blood sample was requested from all participants in the NHS, and 32 826 women provided one. Similarly, between 1993 and 1995, a blood sample was provided by 18 225 men in the HPFS. Participants who provided blood samples were similar to those who did not. In the NHS, among women without CVD or cancer before 1990, we identified 350 women who had a nonfatal myocardial infarction or fatal CHD between the date of blood drawing and June 2004. In the HPFS, we identified 425 men initially free of CVD who had a nonfatal myocardial infarction or fatal CHD between the date of blood draw and the return of the 2004 questionnaire. Using risk‐set sampling,^25^ we randomly selected controls in a 1:1 ratio who were matched for age (±1 year), smoking status (never, past, or current), and date of blood sampling (±2 months) from the participants who were free of CVD at the time CHD was diagnosed in the case patients. Within the NHS cohort, an additional matching criterion was fasting status at the time of blood sampling.

### Assessment of CHD

Study physicians who were unaware of the participant's exposure status confirmed the diagnosis of myocardial infarction on the basis of the criteria of the World Health Organization. Deaths were identified from state vital records and the National Death Index or reported by the participant's next of kin or the postal system. Fatal CHD was confirmed by an examination of hospital or autopsy records, by the listing of CHD as the cause of death on the death certificate, if CHD was the underlying and most plausible cause, and if evidence of previous CHD was available.

### Assessment of Other Factors

Medical history, lifestyle, and demographic information was derived from the questionnaire administered in 1990 to women and 1994 to men, with missing information substituted from previous questionnaires. Physical activity was expressed in terms of metabolic equivalent (MET)‐hours. The questionnaires and the validity and reproducibility of measurements have been described previously.^21–24^

### Measurement of Lipid and Apolipoprotein Levels

Blood samples from women were collected in heparin‐treated tubes and samples from men in EDTA‐treated tubes. The tubes were placed on ice packs, shipped in Styrofoam containers by overnight courier, centrifuged, divided into aliquots, and stored in liquid‐nitrogen freezers (−130°C or colder). One vial containing 0.6 mL (NHS) or 0.5 mL (HPFS) of frozen plasma was shipped to the lipoprotein laboratory at the Harvard School of Public Health for analysis of lipoprotein types. Study samples were sent to the laboratory for analysis in randomly ordered batches, and the laboratory personnel were unaware of a sample's case–control status. The entire plasma sample was thawed, filtered, and incubated overnight with affinity‐purified anti–apoC‐III antibodies (Academy Biomedical) bound to Sepharose 4B resin, as previously described and validated.^3,26^ The unbound lipoproteins that did not contain apoC‐III were collected by gravity flow, and the bound lipoproteins that contained apoC‐III were eluted with 3 mol/L sodium thiocyanate. The efficiency of the apoC‐III immunoaffinity separation (percentage of ligand removed from plasma by the resin) was 99%. VLDL (density below 1.006 g/mL) and LDL (density between 1.006 and 1.063 g/mL) were isolated from plasma with and without apoC‐III by ultracentrifugation.^27^ We used the 1.006 g/mL lower cutoff for LDL to include IDL in our analysis. A small amount of large‐size lipoprotein (a) [Lp(a)] could have been included in this density range. We note here that apoB‐containing remnants of metabolism of triglyceride‐rich VLDL are contained throughout the density interval 1.006 to 1.063 as directly determined by tracer kinetic studies.^4^ ApoB, apoE, and apoC‐III were measured in the lipoprotein fractions by enzyme‐linked immunosorbent assay, and cholesterol and triglycerides were measured by enzymatic methods (Infinity Kit; Thermo Fischer Scientific).

As mentioned in the introduction, we did not measure apoE in LDL without apoC‐III because in previous studies^3–4^ we have found the concentration of this lipoprotein type (LDL with apoE and without apoC‐III) to be negligible or undetectable in most individuals.

The study protocol was approved by the institutional review board of the Brigham and Women's Hospital and the Human Subjects Committee Review Board of the Harvard School of Public Health. After excluding participants with missing data for apoB in one or more of the LDL and VLDL fractions, the dataset consisted of 1476 participants (322 female and 418 male cases and 322 female and 418 male controls). The cases were identified and the controls selected in 2‐year cycles. Two men and 2 women selected as controls in one cycle later developed CHD and were selected in a subsequent cycle, this time as cases. For this reason, the study sample contains 1476 and not 1480 individuals. For these 4 participants, we used the same baseline measurements in the pair where they were cases and in the pair where they were controls. Our main exposure was the content of apoE in LDL with apoC‐III, measured as the number of apoE molecules per LDL particle (apoE:apoB molar ratio).

### Statistical Analysis

Apolipoprotein measurements were divided into quintiles, from the lowest to highest levels, on the basis of the sex‐specific distributions among the controls. We analyzed the association between apolipoprotein levels and the risk of CHD using conditional logistic regression. With risk‐set sampling, the odds ratio derived from the logistic regression directly estimates the hazard ratio and, thus, the relative risk.^25^ We analyzed our main exposure in 3 logistic models: model 1 conditioned on matching factors only; model 2 with additional adjustment for parental history of CHD before the age of 60 years, alcohol intake, and history of hypertension at baseline; and model 3 with adjustment for all the variables in model 2 plus body mass index and history of diabetes at baseline. In additional models, we also adjusted for the plasma LDL cholesterol concentration and by plasma concentrations of C‐reactive protein (CRP). Baseline was defined as the year in which blood was drawn. To test for linear trend, we used the median levels of apolipoprotein measurements in the control quintile categories as a continuous variable. We analyzed all quintiles for all variables, but for ease of reading in Table 2 we display only the relative risk for quintile 5 compared with quintile 1 and the *P* value for trend across quintiles. All *P* values are 2‐tailed, and *P* values <0.05 were considered to indicate statistical significance. All analyses were performed with the use of SAS software, version 9.1 (SAS Institute).

## Results

The average content of apoE (as reflected by the apoE:apoB molar ratio) in LDL with apoC‐III was significantly lower in cases than in matched controls of both sexes (Table 1). Meanwhile, the apoE content of VLDL with apoC‐III was not different in cases and controls.

**Table 1.** Baseline Characteristics of the Study Sample

Characteristic	Women*			Men*		
	Cases (n=322)	Controls (n=322)	*P* Value*	Cases (n=418)	Controls (n=418)	*P* Value*
Age, y	60±6	60±6	—	64.5±8	64.5±8	—
Current smoker, %	26	26	—	9	8	—
Body mass index, kg/m^2^	26.7±5.6	25.4±4.3	0.001	26.1±3.2	25.5±3.5	0.014
Parental history of CHD before age 60 y, %	20	15	0.073	15	12	0.26
Postmenopausal, %	91	88	0.30	—	—	—
Postmenopausal hormone therapy among postmenopausal women, %	33	35	0.55	—	—	—
Aspirin use, %*	26	30	0.28	40	33	0.035
History of hypertension, %	54	32	<0.001	37	28	0.005
History of diabetes, %	17	7	<0.001	9	3	<0.001
Alcohol consumption, g/d						
Median	0.9	1.1	0.17	4.5	7.5	0.006
Quartile 1 to quartile 3	0 to 4.7	0 to 6.4	—	0 to 15	1.0 to 18.3	—
Total cholesterol, mmol/L	6.1±1.2	6±1.2	0.20	5.6±1	5.4±0.9	0.002
LDL cholesterol, mmol/L	3.8±1	3.7±1	0.077	3.5±0.9	3.3±0.8	<0.001
HDL cholesterol, mmol/L	1.3±0.4	1.5±0.4	<0.001	1.1±0.3	1.2±0.3	<0.001
Total:HDL cholesterol ratio	3.7±1.5	3.4±1.6	0.08	3.7±1.1	3.4±1.4	0.002
Triglycerides (mmol/L)	1.6±1.1	1.3±0.7	<0.001	1.5±0.9	1.3±0.8	<0.001
C‐reactive protein, mg/L						
Median	0.51	0.38	0.34	0.34	0.26	0.86
Quartile 1 to quartile 3 (interquartile range)	0.20 to 1.50	0.14 to 0.96	—	0.13 to 1.01	0.09 to 0.86	—
Total plasma apoB, g/L	0.92±0.28	0.88±0.31	0.14	0.97±0.28	0.90±0.26	<0.001
apoE in VLDL with apoCIII, g/L	0.0046±0.0039	0.0040±0.0032	0.075	0.013±0.009	0.012±0.008	0.08
apoE in LDL with apoCIII, g/L	0.0091±0.008	0.0089±0.0065	0.80	0.0093±0.0064	0.0089±0.0062	0.30
apoE in HDL, g/L	0.0094	0.0086	0.58	0.0069	0.0067	0.48
apoE:apoB molar ratio in VLDL with apoC‐III	7.8±7.0	9.0±9.1	0.093	10.6±7.0	11.1±7.9	0.32
apoE:apoB molar ratio in LDL with apoC‐III	2.0±1.3	2.4±1.6	0.001	1.3±0.9	1.5±0.9	0.044
apoE:apoCIII molar ratio in VLDL	0.09±0.14	0.09±0.11	0.99	0.15±0.12	0.16±0.13	0.83
apoE:apoCIII molar ratio in LDL	0.12±0.17	0.13±0.16	0.42	0.10±0.08	0.13±0.52	0.24

Matching criteria were age, smoking status, and date of blood sampling; among women, additional matching criteria included fasting status at the time of blood sampling. The ±values are mean±SD. The body mass index is the weight in kilograms divided by the square of the height in meters. CHD indicates coronary heart disease; LDL, low‐density lipoprotein; HDL, high‐density lipoprotein; VLDL, very low‐density lipoprotein; apoE, apolipoprotein E; apoB, apolipoprotein B; apoC‐III, apolipoprotein C‐III.

* Data on women are from the Nurses' Health Study and include 14 years of follow‐up, and data on men are from the Health Professionals Follow‐up Study and include 10 years of follow‐up.

* *P* values for the difference between patients and controls (unadjusted) were determined by Student *t* test for variables expressed as mean±SD values, by Wilcoxon's rank‐sum test for variables expressed as median values, and by the χ^2^ test for variables expressed as percentages.

* Current aspirin use was defined as every 1 to 4 days per week for women and as 2 or more times per week for men.

**Figure 2.**
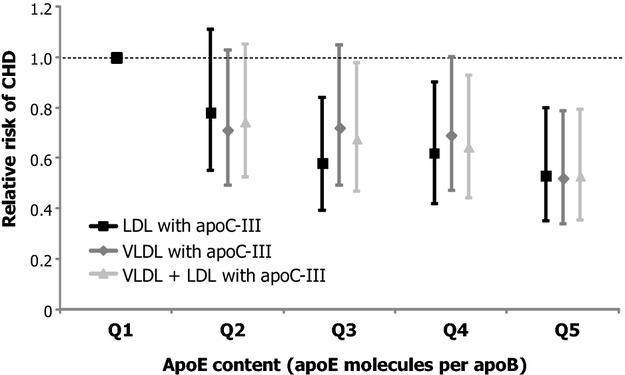
Relative risks of CHD according to apoE contents of VLDL, LDL, or VLDL+LDL (separate models for each fraction). Median apoE contents for each quintile are shown in Figure S1. All risks shown in this figure are derived from model 3 (Table 2), conditioned on matching factors and adjusted for parental history of CHD before the age of 60 years, alcohol intake, history of hypertension at baseline, body mass index, and history of diabetes at baseline. Points represent relative risks; error bars represent 95% CIs. CHD indicates coronary heart disease; VLDL, very low‐density lipoprotein; LDL, low‐density lipoprotein; apoE, apolipoprotein E; apoC‐III, apolipoprotein C‐III.

In these cohorts, we have found the association of LDL with apoC‐III with CHD to be markedly and significantly greater than that of LDL without apoC‐III.^20^ In the present study, the primary result is that a higher apoE content per each LDL with apoC‐III (apoE content per particle) predicted a markedly lower risk of CHD (relative risk for top versus bottom quintile 0.45, 95% CI 0.31 to 0.64), which continued to be significant after multivariable adjustment (Table 2 and Figure 2).

**Table 2.** Relative risks of CHD by quintiles of apoE content in LDL, VLDL, or VLDL+LDL with apoC‐III

	Relative Risk for Q5 vs Q1	*P* Value for Trend
apoE:apoB molar ratio in VLDL with apoC‐III		
Model 1	0.50 (0.35 to 0.72)	<0.001
Model 2	0.48 (0.32 to 0.72)	0.002
Model 3	0.52 (0.34 to 0.79)	0.005
Model 3+plasma LDL cholesterol	0.61 (0.40 to 0.94)	0.040
Model 3+plasma HDL cholesterol	0.61 (0.40 to 0.94)	0.048
Model 3+plasma triglycerides	0.60 (0.39 to 0.92)	0.030
Model 3+plasma C‐reactive protein	0.51 (0.34 to 0.78)	0.005
apoE:apoB molar ratio in LDL with apoC‐III		
Model 1	0.45 (0.31 to 0.64)	<0.001
Model 2	0.47 (0.32 to 0.70)	<0.001
Model 3	0.53 (0.35 to 0.80)	0.002
Model 3+plasma LDL cholesterol	0.64 (0.42 to 0.97)	0.041
Model 3+plasma HDL cholesterol	0.65 (0.43 to 1.00)	0.038
Model 3+plasma triglycerides	0.69 (0.44 to 1.08)	0.09
Model 3+plasma C‐reactive protein	0.53 (0.35 to 0.80)	0.002
apoE:apoB molar ratio in VLDL+LDL with apoC‐III		
Model 1	0.43 (0.30 to 0.62)	<0.001
Model 2	0.48 (0.32 to 0.71)	<0.001
Model 3	0.53 (0.35 to 0.79)	0.002
Model 3+plasma LDL cholesterol	0.63 (0.41 to 0.96)	0.05
Model 3+plasma HDL cholesterol	0.60 (0.40 to 0.90)	0.018
Model 3+plasma triglycerides	0.63 (0.41 to 0.95)	0.034
Model 3+plasma C‐reactive protein	0.50 (0.33 to 0.75)	0.001

Model 1 is conditioned on matching factors only; model 2 is additionally adjusted for parental history of CHD before the age of 60 years, alcohol intake, and history of hypertension at baseline. Model 3 is adjusted for everything in model 2 plus body mass index and history of diabetes at baseline. Relative risks and 95% CIs are given for the highest quintile compared with the lowest quintile of each variable. CHD indicates coronary heart disease; LDL, low‐density lipoprotein; HDL, high‐density lipoprotein; VLDL, very‐low‐density lipoprotein; apoE, apolipoprotein E; apoB, apolipoprotein B; apoC‐III, apolipoprotein C‐III; Q, quintile.

The apoE content of VLDL with apoC‐III, expressed as the apoE:apoB molar ratio in this fraction, also showed a strong negative association with CHD (relative risk for top versus bottom quintile 0.50, 95% CI 0.35 to 0.72; *P* for trend <0.001) that persisted after additional multivariable adjustment in models 2 and 3 (Table 2 and Figure 2). The results in terms of risk estimates were virtually identical when the HPFS and NHS cohorts were analyzed separately (Figure S1).

To explore the influence of apoE on all atherogenic (ie, non‐HDL) lipoproteins simultaneously, we analyzed the relative risks of CHD by quintiles of apoE content in VLDL+LDL. A higher content of apoE in VLDL+LDL with apoC‐III was associated with a markedly lower risk of CHD (relative risk for top versus bottom quintile 0.43, 95% CI 0.30 to 0.62; *P* for trend <0.001), and apoE in VLDL+LDL with apoC‐III continued to show a protective association after adjustment for major cardiovascular risk factors, body mass index, and personal history of diabetes (relative risk 0.53, 95% CI 0.35 to 0.79; *P* for trend 0.002) (Table 2 and Figure 2).

**Figure 3.**
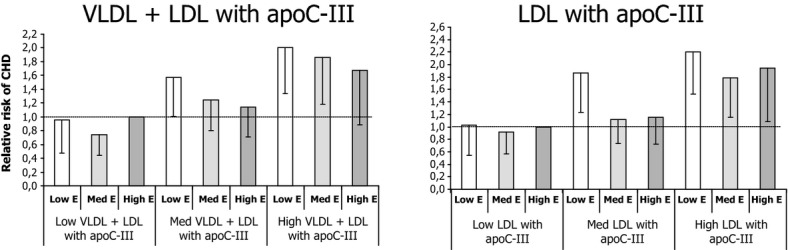
Relative risks of CHD by joint tertiles of apoE content (apoE:apoB molar ratio) and apoB concentration of VLDL+LDL with apoC‐III (left) or LDL with apoC‐III (right). Bars represent relative risks for each joint tertile compared with the reference category (low VLDL+LDL apoC‐III concentration—high apoE/B; or low LDL apoC‐III concentration—high apoE/B). CHD indicates coronary heart disease; VLDL, very low‐density lipoprotein; LDL, low‐density lipoprotein; apoE, apolipoprotein E; apoC‐III, apolipoprotein C‐III; Low E, lowest tertile of apoE content; Med E, medium tertile of apoE content; High E, highest tertile of apoE content.

Given that plasma concentrations of both VLDL and LDL with apoC‐III have been found to predict cardiovascular events,^20^ we explored whether the association between the concentration of this type of lipoproteins (expressed as the apoB concentration in these fractions) and CHD would be modulated by their content of apoE. The highest risk was associated with a high concentration of VLDL+LDL apoC‐III and a low content of apoE in this lipoprotein type or with a high concentration of LDL apoC‐III and a low content of apoE (Figure 3). A higher apoE content was associated with lower risk across the second and third tertiles of VLDL + LDL with apoC‐III and of LDL with apoC‐III (Figure 3), with no significant interaction (*P* value for interaction=0.36), suggesting that a high content of apoE has the potential to attenuate risk conferred by VLDL and LDL with apoC‐III or LDL with apoC‐III, even when their plasma concentration is high.

Adjustment for plasma LDL cholesterol in the background of multivariable model 3 only slightly attenuated the negative association between apoE content of LDL that has apoC‐III and CHD (relative risk 0.62, 95% CI 0.41 to 0.93) and between apoE content of VLDL that has apoC‐III and CHD (relative risk 0.61, 95% CI 0.40 to 0.93). The same pattern was observed in multivariable models adjusted for plasma HDL cholesterol or plasma triglycerides. The associations for both apoE content of LDL with apoC‐III and apoE content in VLDL with apoC‐III were only slightly attenuated. Given that apoE has been proposed to have anti‐inflammatory properties,^7^ we performed multivariable analyses that included baseline plasma CRP levels. In these models, the apoE content of LDL with apoC‐III (relative risk 0.52, 95% CI 0.34 to 0.78) and the apoE content of VLDL with apoC‐III (relative risk 0.51, 95% CI 0.33 to 0.77), were still inversely associated with CHD (Table 2).

Finally, to explore the impact of LDL apoE content in a scenario of constant LDL concentration, we ran a logistic model that included concentrations of apoB, apoC‐III, and apoE in LDL with apoC‐III. In this model, the apoE content of LDL was still negatively associated with CHD risk (relative risk 0.69, 95% CI 0.45 to 1.07).

## Discussion

The results from this prospective study strongly suggest that the presence of apoE is associated with a lower atherogenicity of LDL that contains apoC‐III and that the abundance of apoE relative to the abundance of LDL with apoC‐III is a protective factor against CHD. The apoE content of VLDL also exhibited a negative association with CHD. The protective effect of apoE in LDL appeared to be independent of plasma LDL cholesterol levels and to persist even when apoC‐III levels in LDL are high.

Even though this finding makes sense given the biological antagonism between the 2 apolipoproteins and the well‐documented antiatherogenic properties of apoE,^7^ the influence of apoE in lipoproteins on hard cardiovascular end points is very scantly documented. Our results also emphasize the increasingly recognized importance of the composition and physicochemical properties of lipoproteins as more refined and often more relevant variables in the exploration of pathophysiological mechanisms than the total concentrations of plasma lipids. Even though only 1.5% to 16% of LDL contain apoC‐III,^3^ the idea that apoE can effectively protect against the risk provided by these particles is very appealing if one considers that LDL with apoC‐III are very strongly associated with CHD^19^ and that in fact much of the CHD risk ordinarily attributed to total LDL may be due to this subpopulation that contains apoC‐III.^20^

Prior studies in older adults have found a positive association between total plasma apoE levels and CVD mortality^11^ or the risk of stroke.^12^ From our results, it is apparent that the distribution of total plasma apoE across the different lipoprotein fractions can greatly affect its association with CVD. In this respect, an observational analysis of patients from the Cholesterol and Recurrent Events (CARE) trial found a positive association between apoE in HDL and the risk of coronary events.^13^ This might explain the positive association between plasma apoE and CVD in some studies, given that about 40% to 50% of apoE is found on HDL.^5^ Nevertheless, our results suggest that the relatively minor fraction of plasma apoE that is found in LDL may have a remarkable impact on the risk of CHD. One study in 2 European countries found higher plasma apoE concentrations in VLDL+LDL of myocardial infarction survivors relative to controls who did not survive a coronary event.^28^ In our study, when we exclude the variation in apoB levels by normalizing apoE to the apoB content per particle, apoE was clearly protective in both VLDL and LDL. Also, adjustment for CRP did not diminish the observed effect of apoE, suggesting that the anti‐inflammatory properties of apoE in LDL do not have a major repercussion on its apparent protective ability against CHD.

Finally, a limitation of our study is that we did not evaluate the influence of the apoE ε2/ε3/ε4 polymorphism on the assessed risks, and apoE isoforms have structural differences that may modulate their function in LDL.^29^ Although genetic polymorphisms only partially explain variation in circulating apoE concentrations,^30^ future studies in which the ε2/ε3/ε4 polymorphism is available will help refine our findings. In any event, the evidence we are presenting of an overall protective role for apoE in VLDL and LDL with apoC‐III is a valuable start point for more detailed study.

In summary, apoE in LDL with apoC‐III may mitigate its atherogenicity and association with CHD. This finding should motivate further research on mechanisms to enrich LDL in apoE as a potential intervention for the prevention of CHD.
